# Excess mortality and associated factors among people living with HIV initiating highly active antiretroviral therapy in Luzhou, China 2006–2020

**DOI:** 10.1186/s12879-023-08165-4

**Published:** 2023-03-29

**Authors:** Dandan Niu, Ticheng Xiao, Yuanyi Chen, Houlin Tang, Fangfang Chen, Chang Cai, Qianqian Qin, Decai Zhao, Yichen Jin, Shi Wang, Yushan Hou, Zhen Lu, Luoyao Yang, Hong Liu, Dongqin Xie, Huachun Zou, Fan Lyu

**Affiliations:** 1 Division of Epidemiology, National Center for AIDS/STD Control and Prevention, Chinese Center for Disease Control and Prevention, Beijing, China; 2 Luzhou Prefectural Center for Disease Control and Prevention, Sichuan, China; 3 School of Public Health (Shenzhen), Sun Yat-Sen University, Shenzhen, China

**Keywords:** HIV, Highly active antiretroviral therapy, Mortality, China

## Abstract

**Background:**

To estimate crude mortality, excess mortality, and standardized mortality rates (SMR) among people living with HIV (PLHIV) initiating highly active antiretroviral therapy (HAART) in Luzhou, China 2006–2020, and assess associated factors.

**Methods:**

PLHIV initiating HAART in the HIV/AIDS Comprehensive Response Information Management System (CRIMS) in Luzhou, China 2006–2020 were included in the retrospective cohort study. The crude mortality, excess mortality, and SMR were estimated. Multivariable Poisson regression model was used for analyzing risk factors associated with excess mortality rates.

**Results:**

The median age among 11,468 PLHIV initiating HAART was 54.5 years (IQR:43.1–65.2). The excess mortality rate decreased from 1.8 deaths/100 person-years (95% confidence interval [CI]:1.4–2.4) in 2006–2011 to 0.8 deaths/100 person-years (95%CI:0.7–0.9) in 2016–2020. SMR decreased from 5.4 deaths/100 person-years (95%CI:4.3–6.8) to 1.7 deaths/100 person-years (95%CI:1.5–1.8). Males had greater excess mortality with the eHR of 1.6 (95%CI:1.2–2.1) than females. PLHIV with CD4 counts ≥ 500 cells/μL had the eHR of 0.3 (95%CI:0.2–0.5) in comparison to those with CD4 counts < 200 cells/μL. PLHIV with WHO clinical stages III/IV had greater excess mortality with the eHR of 1.4 (95%CI:1.1–1.8). PLHIV with time from diagnosis to HAART initiation ≤ 3 months had the eHR of 0.7 (95%CI:0.5–0.9) compared to those with time ≥ 12 months. PLHIV with initial HAART regimens unchanged and viral suppression had the eHR of 1.9 (95%CI:1.4–2.6) and 0.1 (95%CI:0.0–0.1), respectively.

**Conclusions:**

The excess mortality and SMR among PLHIV initiating HAART in Luzhou, China decreased substantially from 2006 to 2020, but the mortality rate among PLHIV was still higher than general population. PLHIV who were male, with baseline CD4 counts less than 200 cells/μL, WHO clinical stages III/IV, time from diagnosis to HAART initiation ≥ 12 months, initial HAART regimens unchanged, and virological failure had a greater risk of excess deaths. Early and efficient HAART would be significant in reducing excess mortality among PLHIV.

## Introduction

Acquired immunodeficiency syndrome (AIDS) has become a chronic infectious disease that seriously threatens human health on a global scale. According to the Joint United Nations Programme on HIV/AIDS (UNAIDS), 37.7 million people have become infected with human immunodeficiency virus (HIV), and 680,000 people have died from AIDS and AIDS-related causes in 2020 worldwide [1]. The discovery of highly active antiretroviral therapy (HAART) has promoted AIDS to become controllable [2]. For individuals among PLHIV, HAART does inhibit viral replication [3], reduce viral loads [4], increase CD4 + T lymphocytes (CD4) counts [5], and delay disease progression [6]. For population levels, HAART does prevent transmission [7], reduce incidence and mortality, and extend life expectancy [8]. However, mortality among people living with HIV (PLHIV) remains high in several regions, especially resource-limited settings [9].

Since the implementation of National Free Antiretroviral Treatment Program (NFATP) in 2003 in China, the mortality rate among PLHIV has been substantially reduced. Meanwhile, HAART coverage proportion increased from 9.8% in 2005 to 92.9% in 2020 [10, 11]. However, there existed approximately more than 30,000 PLHIV deaths reported each year in China [12].

The reduction in mortality among PLHIV means that the gap in life expectancy between PLHIV and general population was narrowed. Foreign countries have carried out relevant research and found that early antiretroviral therapy can help reduce the gap between them. For example, in California, on the occasion that PLHIV initiated HAART when baseline CD4 counts ≥ 500 cells/μL, the life expectancy gap compared to general population has been narrowed to 7.9 years during 1996–2011 [13]; In Thailand, the life expectancy among PLHIV with baseline CD4 counts ≥ 350 cells/μL was 51.9 (95%CI:51.0–52.9) years, which was close to general population (56.2 years) [14]. However, the gap between life expectancy of PLHIV initiating HAART and general population in China was still unknown [10].

Luzhou was one of the cities in Southwest China and its reported AIDS epidemic has already become a severe public health problem. Its reported incidence rose from 8.50 cases per 100,000 population in 2011 to 49.25 cases per 100,000 population in 2020 [15]. Its HAART coverage and viral suppression proportions were 92.6% and 91.7% in 2019, respectively [16]. The epidemic situation among PLHIV in Luzhou was, to some extent, a microcosm of the AIDS epidemic in China.

This study compared mortality estimates between PLHIV initiating HAART with the general Chinese population between 2006 and 2020 in Luzhou, China. This study estimated the crude mortality, excess mortality, and standardized mortality rates (SMR), and assessed risk factors associated with excess mortality rates. The estimation of excess mortality and SMR among PLHIV was conductive to evaluating the effectiveness of HAART at population levels [17]. More profoundly, this study would provide a reference for further improvement of HAART strategies and rational allocation of health resources.

## Materials and methods

### Study design and data sources

This retrospective cohort study included PLHIV initiating HAART through NFATP in Luzhou, China in 2006–2020. Data were retrieved from the HIV/AIDS Comprehensive Response Information Management System (CRIMS). This unified real-time web-based national HIV/AIDS information system was launched in 2004 by National Centre for AIDS/STD Control and Prevention (NCAIDS) at Chinese Centers for Disease Control and Prevention (China CDC) [18]. This system has created eight data collection subsystems up to now and covered each city in mainland China, which facilitates data collection and management regularly. Since AIDS was classified as Class B notifiable infectious disease, local medical institutions would report cases online through this national system within 24 h once PLHIV were diagnosed.

This study chose to stratify calendar years into four groups (2006–11, 2012–13, 2014–15, and 2016–20), similar to previous studies [19]. Such grouping not only can correspond with the changes of treatment criteria across calendar year, but also preserve adequate sample sizes within strata. Standardized HAART for PLHIV in China initiated in 2003 since the announcement of the national AIDS control policy *Four Frees and One Care*. The HAART policy remained almost unchanged from 2006 to 2011, and was adjusted frequently during 2012–2020. The enrolment criteria for HAART among PLHIV was initially adjusted from CD4 counts below 200 cells/μL in 2006–2011 to below 350 cells/μL in 2012–2013, below 500 cells/μL in 2014–2015, and no limits in 2016–2020 [19].

### Participants

PLHIV initiating HAART between 1 January 2006 and 31 December 2020 in Luzhou, China in the CRIMS were included, and participants were in line with the NFATP criteria. PLHIV lacking information during the research period were excluded, including WHO clinical stages (*n* = 2), marital status (*n* = 8), and CD4 counts (*n* = 173). The proportions of missing data were quite low (< 2%) and the benefits of multiple imputation were negligible, thus the rest of data could represent overall characteristics.

PLHIV aged 15 years or above when initiating HAART were included in this study. For each participant, the time of follow-up started on date of initiating HAART, and ended on date of HIV-related death or censoring. PLHIV were censored either at the date of withdrawal from NFATP or 31 December 2021, whichever occurred first. For participants loss to follow-up, we viewed the date of last visit as the censoring date.

Baseline information when initiating HAART among PLHIV was collected, including age, sex, marital status, educational level, infection routes, the dates of HIV diagnosis and HAART, the date of death and causes, baseline CD4 counts and viral loads, WHO clinical stages, and HAART regimens. To guarantee data quality, China CDC would randomly select twelve provinces for comprehensive assessment of the quality of collected information each year. Access to surveillance system registration was restricted to trained staff assigned by the local CDC with passwords and encryption keys, and all identified individual information was coded anonymously, all measures designed to protect the privacy of PLHIV. The data were obtained complied with relevant data protection and privacy regulations and individual identifiers were removed. This study was granted a waiver of consent and an exemption status by the Institutional Review Board of National Center for AIDS/STD Control and Prevention, Chinese Center for Disease Control and Prevention (X220314674), given that all data were deidentified and analyzed anonymously. All methods were performed in accordance with the Declaration of Helsinki.

### Statistical analysis

The baseline characteristics among PLHIV initiating HAART by calendar year were analyzed by SPSS version 23.0. Continuous variables were described with median (interquartile range, IQR) and categorical variables with frequencies and proportions. Pearson χ^2^ tests were used for the comparison of different categories.

Crude mortality rates were calculated through number of AIDS-related deaths initiating HAART dividing by person-years at risk in 2006–2020. The formula was: crude mortality rate = observed number of deaths/ person-years at follow-up. Its 95% confidence intervals (95%CIs) were calculated by the formula:$95\%\mathrm{ for R} =\mathrm{Exp}\left[{\mathrm{In}\left(\mathrm{R}\right) \pm 1.96}^{*}\frac{1}{\sqrt{n}}\right]$, where R was crude mortality rate and n was the number of HIV-related deaths among PLHIV initiating HAART [17]. Person-years referred to the time interval from initiating HAART to the date of death or censoring.

Excess mortality rates were calculated as the difference between observed number of deaths in this study and expected number of deaths estimated from general population. The formula was: excess mortality rate = (observed number of deaths-expected number of deaths)/person-years at follow-up. The number of expected deaths was estimated by applying the mortality of the general population in Western China to the study participants in Luzhou, China from 2006–2020. Participants were matched to the general population on age (by 5 year age group), sex, and calendar year. Mortality data for the general population in Western China were obtained from *Data Set of National Mortality Surveillance, 2006–2020* [20]. SMR was calculated as the rate of the observed number of deaths in this study to the expected number of deaths from general population. The formula was: SMR = observed number of deaths/expected number of deaths. 95%CIs were calculated for both excess mortality and SMR.

Crude mortality, excess mortality, and SMR were estimated within strata defined by calendar year. Further estimates of different categories were done according to age, sex, marital status, educational level, infection routes, WHO clinical stages, CD4 counts, time from diagnosis to HAART initiation, initial HAART regimens, HAART regimens transition, and viral suppression. According to WHO criteria, it was considered to reach viral suppression if viral loads after 3–6 months of HAART were less than 1000 copies/ml [21].

The risk factors of excess mortality were assessed through multivariable Poisson regression model [22]. In relative survival model, observed number of deaths in each stratum of PLHIV was modeled with a Poisson process and we used the expected number of deaths in each stratum as an offset. The excess mortality rate was assumed as a piecewise constant hazard each year after initiating HAART. Excess hazard ratios (eHRs) and associated 95%CIs were obtained with a Poisson error structure. We examined changes in relative survival across calendar year adjusted by age, sex, marital status, educational level, infection routes, CD4 counts, WHO clinical stages, time from diagnosis to HAART initiation, initial HAART regimens, HAART regimen transition, and viral suppression. The interpretation of eHR in relative survival model was similar to to that of the hazard ratio in the familiar Cox proportional hazards regression model. For example, an eHR of 0.80 for females relative to males would indicate that females have a 20% lower risk of death compared to males [23]. Hypothesis testing was 2-sided and an alpha of 0.05 was used to indicate a statistically significant difference. All analyses were performed using SAS (version 9.4).

## Results

### Demographic characteristics

A total of 11,468 PLHIV initiating HAART between 2006 and 2020 in Luzhou, China was included in this analysis (Table 1). The median age was 54.5 years old (IQR:43.1–65.2), and the median baseline CD4 counts were 273 cells/μL (IQR:167–395). The number of PLHIV on HAART increased over time. PLHIV on HAART were more likely to be 45–54 years, males, living alone, educated in middle school, and infected by heterosexual contact. The proportion of PLHIV who initiated HAART at WHO clinical stages III/IV increased from 5.1% in 2006–2011 to 26.6% in 2016–2020. PLHIV on HAART with TDF + 3TC + EFV/NVP, HAART regimens unchanged, and viral suppression accounted for 91.2%, 85.9%, and 82.1%, respectively.

**Table 1:** Characteristics Among 11,468 PLHIV Initiating HAART in Luzhou, China 2006–2020

Variables	Groups	Total	2006–2011	2012–2013	2014–2015	2016–2020	*P* value
		**(*N* = 11,468)**	**(*N* = 372)**	**(*N* = 679)**	**(*N* = 1252)**	**(*N* = 9165)**	
Age (years)	15–24	5611 (48.9)	4907 (53.5)	429 (34.3)	206 (30.3)	69 (18.5)	< 0.0001
	25–34	2571 (22.4)	2115 (23.1)	234 (18.7)	143 (21.1)	79 (21.3)	
	35–44	1526 (13.3)	1017 (11.1)	247 (19.7)	169 (24.9)	93 (25.0)	
	45–54	1135 (9.9)	709 (7.7)	223 (17.8)	109 (16.0)	94 (25.3)	
	≥ 55	625 (5.4)	417 (4.6)	119 (9.5)	52 (7.7)	37 (9.9)	
Sex	Female	3172 (27.7)	2474 (27.0)	364 (29.1)	219 (32.3)	115 (30.9)	0.006
	Male	8296 (72.3)	6691 (73.0)	888 (70.9)	460 (67.7)	257 (69.1)	
Marital status	Living alone	5972 (52.1)	4634 (50.6)	721 (57.6)	379 (55.8)	238 (52.1)	< 0.0001
	Married	5496 (47.9)	4531 (49.4)	531 (42.4)	300 (44.2)	134 (47.9)	
Educational level	No schooling	1624 (14.2)	1434 (15.6)	125 (10.0)	50 (7.4)	15 (4.0)	< 0.0001
	Primary school	5514 (48.1)	4723 (51.5)	438 (35.0)	245 (36.1)	108 (29.0)	
	Middle school	2824 (24.6)	2004 (21.9)	409 (32.6)	259 (38.1)	152 (40.9)	
	High school or above	1506 (13.1)	1004 (11.0)	280 (22.4)	125 (18.4)	97 (26.1)	
Infection routes	Sex between men	821 (7.2)	53 (6.5)	62 (7.6)	165 (20.1)	541 (65.9)	< 0.0001
	Other	83 (0.7)	14 (16.9)	12 (14.5)	16 (19.3)	41 (49.4)	
	Heterosexual contact	10,564 (92.1)	305 (2.9)	605 (5.7)	1071 (10.1)	8583 (81.2)	
CD4 counts (cells/μL)	≥ 500	1508 (13.1)	1311 (14.3)	148 (11.8)	34 (5.0)	15 (4.0)	< 0.0001
	350–499	2275 (19.8)	1798 (19.6)	328 (26.2)	96 (14.1)	53 (14.2)	
	200–349	3967 (34.6)	3218 (35.1)	380 (30.4)	236 (34.8)	133 (35.8)	
	0–199	3718 (32.4)	2838 (31.0)	396 (31.6)	313 (46.1)	171 (46.0)	
WHO clinical stages	I/ II	10,600 (92.4)	8700 (94.9)	1091 (87.1)	536 (78.9)	273 (73.4)	< 0.0001
	III/IV	868 (7.6)	465 (5.1)	161 (12.9)	143 (21.1)	99 (26.6)	
Time from diagnosis to HAART initiation	≤ 3 months	8909 (77.7)	175 (2.0)	428 (4.8)	765 (8.6)	7541 (84.6)	< 0.0001
	4–12 months	1268 (11.1)	103 (8.1)	131 (10.3)	218 (17.2)	816 (64.4)	
	≥ 12 months	1291 (11.3)	94 (7.3)	120 (9.3)	269 (20.8)	808 (62.6)	
Initial HAART regimens	TDF + 3TC + EFV/NVP	9243 (80.6)	2 (0.0)	101 (1.1)	714 (7.7)	8426 (91.2)	< 0.0001
	AZT/D4T + 3TC + EFV/NVP	1950 (17.0)	357 (18.3)	566 (29.0)	520 (26.7)	507 (26.0)	
	Other	275 (2.4)	13 (4.7)	12 (4.4)	18 (6.5)	232 (84.4)	
ART regimen transition	Yes	2025 (17.7)	202 (10.0)	353 (17.4)	421 (20.8)	1049 (51.8)	< 0.0001
	No	9443 (82.3)	170 (1.8)	326 (3.5)	831 (8.8)	8116 (85.9)	
Viral suppression	Yes	8596 (75.0)	247 (2.9)	431 (5.0)	863 (10.0)	7055 (82.1)	< 0.0001
	No	1602 (14.0)	76 (4.7)	152 (9.5)	250 (15.6)	1124 (70.2)	
	Unknown	1270 (11.0)	49 (3.9)	96 (7.6)	139 (10.9)	986 (77.6)	

Abbreviations: *TDF* Tenofovir, *3TC* Lamivudine, *EFV* Efavirenz, *NVP* Nevirapine, *AZT* Zidovudine, *D4T* Stavudine, *N* Number

### Observed mortality, excess mortality, and standardized mortality rates

**Fig. 1:**
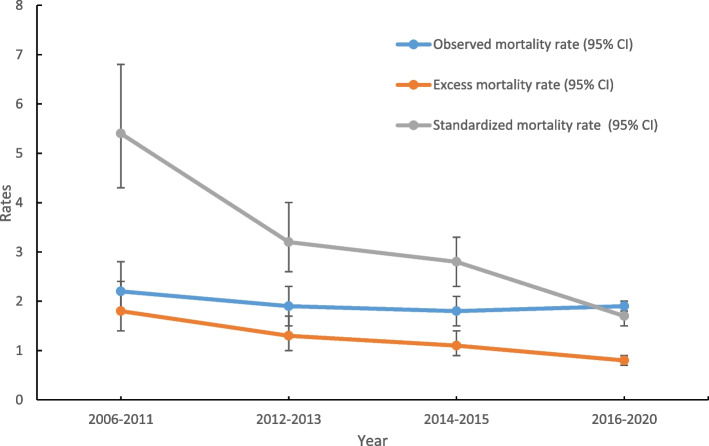
Observed Mortality, Excess Mortality, and Standardized Mortality Rates Among 11,468 PLHIV Initiating HAART in Luzhou, China 2006–2020. Notes: All observed mortality and excess mortality rates (95%CI) were per 100 person-years

The total follow-up time was 39,917.2 person-years, and median follow-up period was 2.8 years (IQR:1.8–4.5). A total of 754 PLHIV (6.6%) died. The overall mortality rate was 1.9 deaths/100 person-years (95%CI:1.7–2.0), and decreased from 2.2 deaths/100 person-years (95%CI:1.8–2.8) in 2006–2011 to 1.9 deaths/100 person-years (95%CI:1.7–2.0) in 2016–2020 (Fig. 1).

The overall excess mortality rate was 0.9 deaths/100 person-years (95%CI:0.8–1.0), and reduced from 1.8 deaths/100 person-years (95%CI:1.4–2.4) in 2006–2011 to 0.8 deaths/100 person-years (95%CI:0.7–0.9) in 2016–2020 (Fig. 1). The overall reductions in excess mortality rates across calendar years among PLHIV initiating HAART were evident in strata of age, sex, marital status, educational level, infection routes, CD4 counts, initial HAART regimens, HAART regimen transition, and viral suppression, except advanced WHO clinical stage and time from diagnosis to HAART initiation ≥ 12 months (Table 2). The most dramatic reductions in excess mortality rates across calendar year occurred among PLHIV with baseline CD4 counts 200–349 cells/μL and married. The excess mortality rate among PLHIV with baseline CD4 counts 200–349 cells/μL decreased from 1.2 (95%CI:0.7–2.0) in 2006–2011 to 0.1 (95%CI:0.1–0.2) in 2016–2020. Among PLHIV married, the excess mortality rate decreased from 2.5 (95%CI:1.8–3.7) in 2006–2011 to 0.3 (95%CI:0.2–0.4) in 2016–2020.

**Table 2:** Excess Mortality Rates Among 11,468 PLHIV Initiating HAART Stratified by Categories in Luzhou, China 2006–2020

Variables	Groups	2006–2011	2012–2013	2014–2015	2016–2020
Age (years)	15–24	1.7 (0.7–4.0)	N/A	1.2 (0.6–2.4)	0.6 (0.3–1.2)
	25–34	0.8 (0.4–1.6)	0.7 (0.3–1.5)	0.6 (0.3–1.2)	0.6 (0.3–1.0)
	35–44	1.9 (1.2–3.1)	1.6 (1.0–2.6)	0.8 (0.5–1.5)	1.1 (0.8–1.5)
	45–54	2.4 (1.5–3.8)	1.7 (1.0–2.7)	1.2 (0.8–2.0)	0.8 (0.6–1.1)
	≥ 55	3.0 (1.8–5.1)	1.6 (1.0–2.5)	1.6 (1.1–2.2)	0.7 (0.6–0.9)
Sex	Female	0.6 (0.3–1.2)	0.3 (0.1–0.7)	0.5 (0.3–0.9)	N/A
	Male	2.5 (1.9–3.2)	1.9 (1.4–2.4)	1.4 (1.1–1.8)	1.2 (1.0–1.4)
Marital status	Living alone	1.5 (1.0–2.1)	1.4 (1.0–1.9)	1.5 (1.1–1.9)	1.2 (1.0–1.4)
	Married	2.5 (1.8–3.7)	1.2 (0.8–1.8)	0.7 (0.5–1.1)	0.3 (0.2–0.4)
Educational level	No schooling	3.6 (1.2–10.6)	2.8 (1.4–5.7)	3.3 (2.1–5.2)	0.9 (0.7–1.3)
	Primary school	2.4 (1.5–3.6)	1.5 (1.0–2.3)	1.0 (0.6–1.4)	0.8 (0.6–0.9)
	Middle school	2.1 (1.5–3.1)	1.3 (0.9–2.0)	1.0 (0.7–1.5)	0.9 (0.7–1.1)
	High school or above	0.8 (0.4–1.6)	0.5 (0.2–1.2)	0.9 (0.5–1.4)	0.4 (0.2–0.7)
Infection routes	Sex between men	0.8 (0.3–2.2)	0.1 (0.0–1.8)	0.9 (0.5–1.7)	0.7 (0.4–1.2)
	Other	3.0 (1.1–8.2)	3.3 (1.0–10.5)	3.1 (1.0–9.9)	3.5 (1.3–9.5)
	Heterosexual contact	2.0 (1.5–2.6)	1.4 (1.1–1.8)	1.1 (0.9–1.5)	0.8 (0.7–0.9)
CD4 counts (cells/μL)	≥ 500	0.8 (0.1–4.9)	1.6 (0.6–4.5)	0.6 (0.2–1.4)	N/A
	350–499	0.1 (0.0–1.5)	0.8 (0.4–1.9)	0.9 (0.5–1.4)	0.1 (0.0–0.2)
	200–349	1.2 (0.7–2.0)	0.4 (0.2–0.9)	0.4 (0.2–0.8)	0.1 (0.1–0.2)
	0–199	3.1 (2.3–4.2)	2.3 (1.7–3.1)	2.5 (1.9–3.3)	2.5 (2.2–2.9)
WHO clinical stages	I/ II	1.7 (1.2–2.3)	0.7 (0.5–1.1)	0.8 (0.6–1.1)	0.6 (0.5–0.7)
	III/IV	2.2 (1.4–3.6)	4.0 (2.9–5.7)	3.5 (2.4–5.1)	3.2 (2.3–4.4)
Time from diagnosis to HAART initiation	≤ 3 months	2.2 (1.6–3.1)	1.1 (0.8–1.5)	1.2 (0.9–1.6)	0.6 (0.5–0.7)
	4–12 months	1.8 (1.1–2.9)	1.5 (0.9–2.5)	0.2 (0.1–0.7)	1.4 (1.0–1.9)
	≥ 12 months	1.2 (0.7–2.3)	2.0 (1.2–3.3)	1.8 (1.2–2.6)	1.7 (1.3–2.3)
Initial HAART regimens	TDF + 3TC + EFV/NVP	N/A	1.2 (0.6–2.3)	1.0 (0.8–1.4)	0.7 (0.6–0.8)
	AZT/D4T + 3TC + EFV/NVP	1.7 (1.3–2.3)	1.4 (1.0–1.8)	1.3 (1.0–1.8)	1.3 (0.8–2.0)
	Other	5.0 (2.1–12.1)	N/A	N/A	2.5 (1.5–4.3)
ART regimen transition	Yes	0.8 (0.5–1.3)	0.2 (0.1–0.5)	0.2 (0.1–0.4)	0.4 (0.2–0.7)
	No	3.5 (2.6–4.7)	3.1 (2.4–4.0)	1.7 (1.4–2.2)	0.8 (0.7–0.9)
Viral suppression	Yes	0.3 (0.1–0.6)	N/A	0.1 (0.1–0.3)	N/A
	No	1.6 (0.8–2.9)	1.9 (1.2–2.9)	1.3 (0.8–2.0)	0.8 (0.6–1.2)
	Missing	56.8 (42.1–76.7)	44.0 (32.8–58.9)	22.2 (17.0–28.8)	20.2 (17.9–22.8)

All excess mortality rates (95%CI) were per 100 person-years

Abbreviations: *N/A* Not applicable, *TDF* Tenofovir, *3TC* Lamivudine, *EFV* Efavirenz, *NVP* Nevirapine, *AZT* Zidovudine, *D4T* Stavudine

The overall SMR was 1.9 (95%CI:1.8–2.1), and decreased from 5.4 (95%CI:4.3–6.8) in 2006–2011 to 1.7 (95%CI:1.5–1.8) in 2016–2020 (Fig. 1). The changes of SMR stratified by PLHIV characteristics were close to excess mortality rates. SMR also showed reductions across the calendar years in strata of sex, marital status, educational level, CD4 counts, WHO clinical stages, time from diagnosis to HAART initiation, initial HAART regimens, HAART regimen transition, and viral suppression, except the 25–34 years old and other infection routes groups (Table 3). The most dramatic reductions in SMR across calendar year occurred among PLHIV married and with initial HAART regimen unchanged. Among PLHIV married, SMR decreased from 7.0 (95%CI:5.0–9.8) in 2006–2011 to 1.3 (95%CI:1.1–1.5) in 2016–2020. SMR among PLHIV with initial HAART regimens unchanged decreased from 7.9 (95%CI:6.0–10.5) in 2006–2011 to 1.7 (95%CI:1.6–1.9) in 2016–2020.

**Table 3:** Standardized Mortality Rates Among 11,468 PLHIV Initiating HAART Stratified by Categories in Luzhou, China 2006–2020

Variables	Groups	2006–2011	2012–2013	2014–2015	2016–2020
Age (years)	15–24	27.4 (11.4–65.9)	N/A	35.6 (17.8–71.2)	19.3 (9.7–38.7)
	25–34	9.6 (4.8–19.2)	10.1 (4.8–21.2)	11.6 (6.2–21.6)	10.5 (6.2–17.8)
	35–44	10.1 (6.3–16.0)	10.2 (6.6–15.7)	7.1 (4.2–12.0)	9.7 (7.0–13.4)
	45–54	6.4 (4.1–9.9)	5.5 (3.6–8.4)	4.3 (2.8–6.5)	3.3 (2.6–4.2)
	≥ 55	2.9 (1.9–4.4)	1.9 (1.4–2.7)	1.9 (1.5–2.4)	1.4 (1.2–1.5)
Sex	Female	2.0 (1.1–3.5)	1.4 (0.9–2.3)	1.5 (1.1–2.2)	0.7 (0.6–0.9)
	Male	8.1 (6.3–10.4)	4.5 (3.6–5.6)	3.6 (3.0–4.4)	2.1 (1.9–2.3)
Marital status	Living alone	4.6 (3.4–6.2)	3.7 (2.8–4.9)	3.4 (2.8–4.2)	2.0 (1.8–2.3)
	Married	7.0 (5.0–9.8)	2.8 (2.0–3.8)	2.0 (1.5–2.7)	1.3 (1.1–1.5)
Educational level	No schooling	2.9 (1.2–6.9)	2.4 (1.4–4.1)	2.5 (1.7–3.5)	1.4 (1.2–1.7)
	Primary school	4.5 (3.1–6.5)	2.9 (2.1–4.0)	2.1 (1.5–2.8)	1.6 (1.4–1.8)
	Middle school	7.7 (5.5–10.9)	4.7 (3.3–6.8)	4.0 (2.8–5.6)	2.6 (2.1–3.2)
	High school or above	6.0 (3.1–11.5)	3.3 (1.6–6.8)	5.1 (3.2–8.1)	2.5 (1.6–3.8)
Infection routes	Sex between men	8.7 (3.6–20.8)	1.6 (0.2–11.7)	15.7 (8.4–29.2)	5.6 (3.3–9.4)
	Other	16.7 (6.3–44.5)	16.7 (5.4–51.7)	23.1 (7.5–71.7)	23 (8.6–61.4)
	Heterosexual contact	5.1 (4.0–6.5)	3.2 (2.6–3.9)	2.5 (2.1–3.0)	1.6 (1.5–1.8)
CD4 counts (cells/μL)	≥ 500	2.4 (0.6–9.6)	4.4 (1.8–10.5)	2.2 (1.1–4.1)	0.9 (0.6–1.3)
	350–499	1.4 (0.3–5.6)	2.3 (1.2–4.3)	2.9 (2.0–4.3)	1.0 (0.8–1.4)
	200–349	3.4 (2.2–5.2)	1.7 (1.1–2.6)	1.5 (1.0–2.2)	1.1 (0.9–1.3)
	0–199	9.7 (7.3–12.8)	5.2 (4.0–6.7)	4.4 (3.5–5.6)	3.0 (2.7–3.4)
WHO clinical stages	I/ II	4.8 (3.7–6.3)	2.3 (1.7–3.0)	2.3 (1.9–2.8)	1.6 (1.4–1.7)
	III/IV	7.9 (5.1–12.1)	7.4 (5.4–10.2)	6.3 (4.4–8.8)	4.1 (3.1–5.5)
Time from diagnosis to HAART initiation	≤ 3 months	6.6 (4.8–9.1)	2.7 (2.0–3.5)	2.5 (2.1–3.1)	1.5 (1.3–1.7)
	4–12 months	4.9 (3.2–7.6)	3.2 (2.1–5.1)	1.4 (0.7–2.7)	2.2 (1.7–2.9)
	≥ 12 months	4.0 (2.4–6.8)	6.9 (4.4–10.8)	5.4 (3.8–7.6)	3.4 (2.6–4.3)
Initial HAART regimens	TDF + 3TC + EFV/NVP	N/A	3.5 (2.0–6.4)	2.3 (1.9–2.9)	1.6 (1.5–1.8)
	AZT/D4T + 3TC + EFV/NVP	5.1 (4.0–6.5)	3.2 (2.6–4.0)	3.7 (2.8–4.8)	2.0 (1.4–2.8)
	Other	54.1 (22.5–129.9)	N/A	N/A	2.7 (1.8–4.1)
ART regimen transition	Yes	3.2 (2.1–4.8)	1.4 (0.9–2.1)	1.3 (0.8–1.9)	1.3 (1.0–1.8)
	No	7.9 (6.0–10.5)	6.2 (4.9–8.0)	3.6 (3.0–4.3)	1.7 (1.6–1.9)
Viral suppression	Yes	1.8 (1.1–2.9)	0.8 (0.5–1.3)	1.2 (0.9–1.7)	0.6 (0.5–0.7)
	No	3.6 (2.1–6.1)	4.5 (3.0–6.6)	2.7 (1.9–3.9)	1.6 (1.3–2.0)
	Missing	82.7 (61.3–111.5)	40.7 (30.5–54.3)	17.9 (13.9–23.1)	12.4 (11.0–13.9)

Abbreviations: *N/A* Not applicable, *TDF* Tenofovir, *3TC* Lamivudine, *EFV* Efavirenz, *NVP* Nevirapine, *AZT* Zidovudine, *D4T* Stavudine

### Risk factors of excess mortality rates among 11,468 PLHIV initiating HAART from 2006 to 2020

Adjusted by general population mortality and other PLHIV characteristics, the excess risk of death among PLHIV initiating HAART in 2016–2020 was nearly 60% lower in 2006–2011(Table 4). The excess risk of death among males was higher with an eHR of 1.6 (95%CI:1.2–2.1). The excess risk of death among PLHIV with baseline CD4 counts above 500 cells/μL was nearly 70% lower than PLHIV with CD4 counts below 200 cells/μL. The excess risk of death of PLHIV with WHO clinical stages III/IV was higher with an eHR of 1.4 (95%CI:1.1–1.8). Compared with PLHIV with time from diagnosis to HAART initiation ≥ 12 months, the excess risk of death among PLHIV with time ≤ 3 months reduced by about 30%. PLHIV with changed initial HAART regimens or viral suppression were at lower excess risk of death.

**Table 4:** Adjusted Excess Hazard Ratios Among 11,468 PLHIV Initiating HAART in Luzhou, China 2006–2020

Variables	Groups	eHR (95%CI)	*P* value
Age (years)	15–24	0.9 (0.5–1.5)	0.632
	25–34	0.8 (0.5–1.2)	0.280
	35–44	0.9 (0.7–1.3)	0.689
	45–54	1.0 (0.8–1.3)	0.963
	≥ 55	Ref	
Sex	Male	1.6 (1.2–2.1)	0.001
	Female	Ref	
Marital status	Married	0.6 (0.5–0.8)	0.256
	Ling alone	Ref	
Educational level	High school or above	0.8 (0.5–1.2)	0.248
	Middle school	1.2 (0.9–1.6)	0.321
	Primary school	0.9 (0.7–1.2)	0.376
	No schooling	Ref	
Infection routes	Sex between men	0.7 (0.4–1.3)	0.282
	Other	0.5 (0.2–1.2)	0.141
	Heterosexual contact	Ref	
CD4 counts (cells/μL)	≥ 500	0.3 (0.2–0.5)	< 0.0001
	350–499	0.3 (0.2–0.5)	< 0.0001
	200–349	0.3 (0.2–0.4)	< 0.0001
	0–199	Ref	
WHO clinical stages	III/IV	1.4 (1.1–1.8)	0.004
	I/II		
Year of HAART initiation	2016–2020	0.4 (0.2–0.6)	< 0.0001
	2014–2015	0.4 (0.3–0.6)	< 0.0001
	2012–2013	0.6 (0.4–0.9)	0.007
	2006–2011	Ref	
Time from diagnosis to HAART initiation	≤ 3 months	0.7 (0.5–0.9)	0.009
	4–12 months	0.5 (0.3–0.7)	0.001
	≥ 12 months	Ref	
Initial HAART regimens	TDF + 3TC + EFV/NVP	0.8 (0.6–1.1)	0.173
	Other	1.3 (0.7–2.3)	0.367
	AZT/D4T + 3TC + EFV/NVP	Ref	
ART regimen transition	No	1.9 (1.4–2.6)	0.0001
	Yes	Ref	
Viral suppression	Yes	0.1 (0.0–0.1)	< 0.0001
	Missing	12.3 (9.1–16.7)	< 0.0001
	No	Ref	

Abbreviations: *TDF* Tenofovir, *3TC* Lamivudine, *EFV* Efavirenz, *NVP* Nevirapine, *AZT* Zidovudine, *D4T* Stavudine

## Discussion

The overall mortality, excess mortality, and SMR among 11,468 PLHIV initiating HAART in Luzhou, China decreased from 2006–2020, but the mortality rate among participants was still higher than general population. The decreases in excess mortality and SMR across calendar year were relatively consistent within different strata of sex, marital status, educational level, CD4 counts, initial HAART regimens, HAART regimen transition, and viral suppression. Being male, baseline CD4 counts less than 200 cells/μL, WHO clinical stages III/IV, time from diagnosis to HAART initiation ≥ 12 months, unchanged initial HAART regimens, and virological failure were risk factors of excess mortality rates among PLHIV on HAART. This study fills in the comparison of life expectancy between PLHIV initiating HAART and general population and indicates the importance of early and effective treatment. These findings provided precious evidence for further policy making in AIDS prevention and treatment. Methodologically, application of the multivariable Poisson regression model in this study could eliminate the impact of missing data on representativeness and confounding biases.

The overall mortality rates decreased about 15.0%, and both the excess mortality and SMR decreased by over 55.0% from 2006 to 2020. This finding was in line with other studies which have also reported reductions in observed mortality rates in both resource rich and poor areas worldwide [24]. The decrease in mortality may be attributed to the increase in treatment coverage. Data showed that the proportion of HAART coverage in Luzhou increased from 76.35% in 2010–2019 to 92.6% in 2019 [25]. The improvement of treatment coverage cannot be separated from the synchronous increase of the number of PLHIV diagnosed. How to innovate methods to improve diagnosis discovery rate still faces certain challenges.

The estimation of excess mortality and SMR indicated that the mortality rate among PLHIV on HAART was still higher than general population. This finding was consistent with other countries. The excess mortality rates in Africa, Europe and North America were 7.0 deaths/100 person-years [23] and 2.0 deaths/100 person-years [26]. Therefore, it can be inferred that the excess mortality may be related to the level of economic development in different regions. Another reason why the mortality among PLHIV on HAART was still higher than general population may be that the complete viral suppression was not achieved. According to published data [16], the proportion of viral suppression in Luzhou in 2019 was 91.7%, and there was still a certain gap from UNAIDS Fast-Track 95–95–95 target [27]. To further reduce mortality among PLHIV, we need to pay attention to important associated factors, such as treatment adherence [28], drug resistance [29], and viral suppression [24].

The excess mortality and SMR among PLHIV stratified by different categories of demographic and clinical characteristics also showed substantial decrease. These characteristics included marital status, CD4 counts, and initial HAART regimens transition, which may be important reminder of risk factors associated with excess mortality.

Multivariate regression analysis showed sex, baseline CD4 counts, WHO clinical stages, time from diagnosis to HAART initiation, initial HAART regimens transition, and virological failure had an effect on excess mortality rates among PLHIV on HAART. The excess mortality rate among men was higher, which may be related to that women tended to obtain better HAART effect and adherence [30]. Another reason may be that women were inclined to achieve higher CD4 levels [31] and better drug responses due to genes [32]. PLHIV initiating HAART with CD4 counts less than 200 cells/μL, WHO clinical stages III/IV and time from diagnosis to HAART initiation ≥ 12 months had higher risk of death, which was consistent with another result [23]. The reason may be that PLHIV with late HAART initiation had worse HAART effect. Research showed that these characteristics could decrease the function of immune system and extend duration of elevated viral loads [33]. Therefore, early HAART would be significant to avoid occurrence of death.

The excess mortality rates among PLHIV with initial HAART regimens unchanged were higher, and can be explained by the fact that immediate adjustment of ineffective HAART regimens may prevent the occurrence of death. This finding suggested that we should conduct regular drug resistance testing for PLHIV receiving HAART. PLHIV with viral suppression had lower excess risk of death, which was in accordance with another result [34]. This finding showed that PLHIV achieving successful antiretroviral therapy were more likely to have a normal life expectancy.

### Limitations

There are some limitations in this study. PLHIV initiating HAART in Luzhou, China were matched to the general Chinese population just on age, sex, and calendar year and caused weak comparability. There were still other background characteristics that this study failed to consider due to unavailability of information, for example, area of residence. Since there were no reliable local mortality data for the general population in Luzhou, China, this study used data from the National Passive Surveillance System in Western China to make a rough estimate. The reported number of deaths among PLHIV initiating HAART in Luzhou, China based the national passive surveillance system may underestimate the actual number. The estimation of excess mortality rate and SMR in this study was just based on the data from Luzhou, China, thus its generalization was relatively weak. We were also unable to account for drug resistance and treatment adherence due to insufficient data, and further research about the impact of these potential factors on mortality was needed.

## Conclusion

Among 11,468 PLHIV initiating HAART in Luzhou, China, we have observed substantial decreases in excess mortality rates and SMR from 2006 to 2020, but the mortality rate was still higher than that of the general population. Male, baseline CD4 counts less than 200cells/μL, WHO clinical stages III/IV, time from diagnosis to HAART initiation ≥ 12 months, unchanged initial HAART regimens, and virological failure were risk factors of excess mortality rate among PLHIV initiating HAART. These findings provided data support for the formulation of the prevention and control measures of AIDS, and further reduction of excess mortality rate would be achieved if HAART was initiated for PLHIV early and efficiently.

## Data Availability

The datasets used and/or analyzed during the current study were available from the corresponding author on reasonable request.

## References

[1] UNAIDS. FACT SHEET 2021[EB/OL] [Available from: https://www.unaids.org/en/resources/fact-sheet.2021-09-26.

[2] Menéndez-Arias L, Delgado R (2022). Update and latest advances in antiretroviral therapy. Trends Pharmacol Sci.

[3] Shet A, Neogi U, Sahoo PN, De Costa A (2013). Effectiveness of first-line antiretroviral therapy and acquired drug resistance among HIV-1-infected children in India. Pediatr Infect Dis J.

[4] Lu Z, Jiao Y, Li J, Lan G, Lu C, Li X (2018). After 18 months of antiretroviral therapy, total HIV DNA decreases more pronouncedly in patients infected by CRF01_AE than in those infected by subtype B and CRF07_BC. Microbiol immunol.

[5] Cao Z, Li J, Chen H, Song C, Shen Z, Zhou X (2020). Effects of HIV-1 genotype on baseline CD4+ cell count and mortality before and after antiretroviral therapy. Sci Rep.

[6] Lw A, Ekm A, Jem A, Wcm B, An C, Pcs D (2019). Combined estimation of disease progression and retention on antiretroviral therapy among treated individuals with HIV in the USA: a modelling study - ScienceDirect. Lancet HIV.

[7] Tang Z, Lan G, Chen YQ, Zhu Q, Yang X, Shen Z (2015). HIV-1 Treatment-as-prevention: a cohort study analysis of serodiscordant couples in rural Southwest China. Medicine.

[8] Teeraananchai S, Kerr S, Amin J, Ruxrungtham K, Law M (2017). Life expectancy of HIV-positive people after starting combination antiretroviral therapy: a meta-analysis. HIV Med.

[9] Braitstein P, Brinkhof MWG, Dabis F, Schechter M, Boulle A, Miotti P (2006). Mortality of HIV-1-infected patients in the first year of antiretroviral therapy: comparison between low-income and high-income countries. Lancet.

[10] Ding Y, Ma Z, He J, Xu X, He N (2019). Evolving HIV epidemiology in Mainland China: 2009–2018. Curr HIV-AIDS Rep.

[11] Zhang F, Dou Z, Ye M, Yao Z, Yan Z, Zhao D (2011). Effect of earlier initiation of antiretroviral treatment and increased treatment coverage on HIV-related mortality in China: a national observational cohort study. Lancet Infect Dis.

[12] Wu Z, Mcgoogan JM, Detels R (2020). The enigma of the HIV epidemic in China. Clin Infect Dis.

[13] Marcus JL, Chao CR, Leyden WA, Xu L, Quesenberry CPJ, Klein DB (2016). Narrowing the gap in life expectancy between HIV-infected and HIV-uninfected individuals with access to care. JAIDS-J Acq Imm Def.

[14] Teeraananchai S, Chaivooth S, Kerr SJ, Bhakeecheep S, Avihingsanon A, Teeraratkul A (2017). Life expectancy after initiation of combination antiretroviral therapy in Thailand. Antivir Ther.

[15] Ren N, Li Y, Wang R, Zhang W, Chen R, Xiao T (2022). The Distribution of HIV and AIDS cases in Luzhou, China, from 2011 to 2020: Bayesian spatiotemporal analysis. JMIR Public Health Surveill.

[16] Wang LQ, Li SQ, Zhou MH (2021). Analysis of treatment status and causes of untreatment in Luzhou acquired immunodeficiency syndrome treatment management. J Community Med.

[17] Zhu H, Napravnik S, Eron JJ, Cole SR, Ma Y, Wohl DA (2013). Decreasing excess mortality of HIV-infected patients initiating antiretroviral therapy: comparison with mortality in general population in China, 2003–2009. JAIDS-J Acq Imm Def.

[18] Yurong M, Zunyou W, Katharine P, Changhe W, Qianqian Q, Ye M (2010). Development of a unified web-based national HIV/AIDS information system in China. Int J Epidemiol.

[19] Chen F, Cai C, Wang S, Qin Q, Jin Y, Li D (2022). Trends in suicide mortality among people with HIV after diagnosis during 2012–18: a retrospective, national cohort study in China. Lancet HIV.

[20] CCDC. Data set of national mortality surveillance, 2006–2020 [Available from: https://ncncd.chinacdc.cn/xzzq_1/202101/t20210111_223706.htm.

[21] Wung BA, Peter NF, Atashili J, Chocontapiraquive LA, De L, Sarmientolimas CA, et al. Consolidated guidelines on the use of antiretroviral drugs for treating and preventing HIV infection: recommendations for a public health approach. 2nd ed. BMC Health Serv Res. 2016;16:129.

[22] Dickman PW, Sloggett A, Hills M, Hakulinen T (2004). Regression models for relative survival. Stat Med.

[23] Brinkhof M, Boulle A, Weigel R, Messou E, Mathers C, Orrell C (2009). Mortality of HIV-Infected Patients Starting Antiretroviral Therapy in Sub-Saharan Africa: Comparison with HIV-Unrelated Mortality. PLoS Med.

[24] Judd A, Chappell E, Turkova A, Coeur SL, Noguerajulian A, Goetghebuer T (2018). Long-term trends in mortality and AIDS-defining events after combination ART initiation among children and adolescents with perinatal HIV infection in 17 middle- and high-income countries in Europe and Thailand: A cohort study. PLoS Med.

[25] Wu Y, Feng C, Chen H, Guo M (2021). Analysis of survival and its related factors of HIV/AIDS patient in Luzhou,2010-2019. Chin J Dis Control Prev.

[26] Bhaskaran K, Hamouda O, Sannes M, Boufassa F, Porter K (2008). Changes in the risk of death after HIV seroconversion compared with mortality in the general population. JAMA.

[27] Frescura L, Godfrey-Faussett P, Feizzadeh AA, El-Sadr W, Syarif O, Ghys PD (2022). Achieving the 95 95 95 targets for all: A pathway to ending AIDS. PLoS ONE.

[28] Ayalew MB (2017). Mortality and its predictors among hiv infected patients taking antiretroviral treatment in Ethiopia: A systematic review. AIDS Res Treat.

[29] Pinoges L, Schramm B, Poulet E, Balkan S, Pujades-Rodriguez M (2015). Risk factors and mortality associated with resistance to first-line antiretroviral therapy: multicentric cross-sectional and longitudinal analyses. J Acquir Immune Defic Syndr.

[30] Gao D, Zou Z, Dong B, Zhang W, Chen T, Cui W (2019). Secular trends in HIV/AIDS mortality in China from 1990 to 2016: Gender disparities. PLoS ONE.

[31] Makhtar C, Tandakha ND, Moussa S, Aziz DA, Marema F, Alassane DP (2010). Low-level CD4+ T cell activation in HIV-exposed seronegative subjects: influence of gender and condom use. J Infect Dis.

[32] Dou Z, Xu J, Jiao JH, Ye M, Zhang F (2011). Gender difference in 2-year mortality and immunological response to ART in an HIV-infected Chinese population, 2006–2008. PLoS ONE.

[33] Nosyk B, Min J, Lima VD, Yip B, Hogg RS, Montaner J (2013). HIV-1 disease progression during highly active antiretroviral therapy: an application using population-level data in British Columbia: 1996–2011. JAIDS-J Acq Imm Def.

[34] May MT, Gompels M, Delpech V, Porter K, Orkin C, Kegg S (2014). Impact on life expectancy of HIV-1 positive individuals of CD4+ cell count and viral load response to antiretroviral therapy. AIDS.

